# Beobachtungsstudie ärztlicher und pflegerischer Aktivitäten in der Notaufnahme

**DOI:** 10.1007/s00063-020-00657-4

**Published:** 2020-02-18

**Authors:** M. Weigl, T. Händl, M. Wehler, A. Schneider

**Affiliations:** 1 Institut und Poliklinik für Arbeits‑, Sozial- und Umweltmedizin, Klinikum der Ludwig-Maximilians-Universität München, Ziemssenstr. 1, 80336 München, Deutschland; 2 Zentrale Notaufnahme und IV. Medizinische Klinik, Universitätsklinikum Augsburg, Augsburg, Deutschland; 3 Institut für Medizinische Soziologie und Rehabilitationswissenschaft, Charité Universitätsmedizin Berlin, Berlin, Deutschland

**Keywords:** Tätigkeiten, Notaufnahme, Versorgungsforschung, Notfallversorgung, Stress, Arbeitszeit, Activities, Emergency room, Health services research, Emergency medicine, Occupational stress, Work schedule

## Abstract

**Hintergrund:**

Systematische und vergleichende Analysen der Tätigkeiten des ärztlichen und pflegerischen Personals in der Notaufnahme fehlen für den deutschsprachigen Bereich.

**Ziel der Arbeit:**

Analyse der Aktivitäten des pflegerischen und ärztlichen Personals einer Notaufnahme sowie der Anteile direkten Patientenkontakts und stündlicher Tätigkeitswechsel.

**Material und Methoden:**

Tätigkeitsanalysen auf Basis teilnehmender Beobachtungen (je 90 min) bei Pflegekräften und Ärzt*innen einer interdisziplinären Notaufnahme eines süddeutschen Krankenhauses der Maximalversorgung. Beobachtete Tätigkeiten wurden anhand eines Klassifikationssystems mitsamt Zeitdauern kodiert. Insgesamt wurden 160 Einzelbeobachtungen (mit einer Gesamtzeit von ca. 240 h) durchgeführt; 99 bei Pflegekräften sowie 61 bei Ärzt*innen.

**Ergebnisse:**

Notaufnahmeärzt*innen arbeiten 30 % ihrer Zeit in direktem Patientenkontakt, Pflegekräfte hingegen 44 %. Für die Einzeltätigkeiten entfielen die größten Zeitanteile ärztlicher Tätigkeit auf Dokumentation und Schriftarbeit (29,3 %), interne Kommunikation mit Personal (16,9 %) sowie mit Patient*innen (13,6 %). Pflegekräfte verwenden die meiste Zeit auf therapeutische und Behandlungsaktivitäten (27,6 %) sowie interne Kommunikation (17,9 %). Diese Tätigkeiten waren stark fragmentiert: Im Durchschnitt erfassten wir 41,3 Einzeltätigkeiten pro Stunde mit einer durchschnittlichen Dauer von 1,5 min. Pflegekräfte hatten signifikant kürzere Tätigkeitsdauern als Ärzt*innen (F[df = 1] = 4,5; *p* = 0,04). Tätigkeitsspezifische Analysen ergaben weitere Professionsunterschiede.

**Diskussion:**

Unsere Ergebnisse liefern erstmalig fundierte Einsichten in die Verteilung und Dauer von ärztlichen sowie pflegerischen Tätigkeiten in der akutmedizinischen Versorgung in der Notaufnahme. Zukünftige Arbeiten sollten sich insbesondere einhergehenden Auswirkungen auf die Leistungsfähigkeit und Beanspruchung des Personals wie auch der Sicherheit und Qualität der Versorgung widmen.

**Zusatzmaterial online:**

Die Online-Version dieses Beitrags (10.1007/s00063-020-00657-4) enthält die Tabelle S1. Beitrag und Zusatzmaterial stehen Ihnen auf www.springermedizin.de zur Verfügung. Bitte geben Sie dort den Beitragstitel in die Suche ein, das Zusatzmaterial finden Sie beim Beitrag unter „Ergänzende Inhalte“.

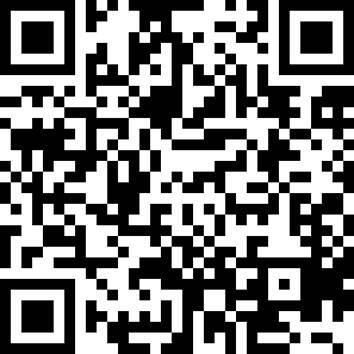

## Abstract (en)

**Background:**

Comprehensive and systematic assessments of nurse and physician activities in the emergency department (ED) are lacking for German-speaking countries.

**Objectives:**

Assessment of work activities of ED nurses and physicians with particular focus on frequencies of direct patient contact as well as rates of activity changes.

**Material and methods:**

We employed standardized assessments of work activities using participant observations (90 min each) among nurses and physicians during their regular shifts. The setting was an interdisciplinary ED of a Southern German academic hospital. Observed activities were classified according to an established system and recorded with time stamps. Overall, 160 observation sessions were conducted (with an observation time of approximately 240 h; 99 among nurses, 61 among physicians).

**Results:**

Physicians spent 30% of their working time in direct patient contact, nurses 44%. Concerning individual activities, the largest proportions of physicians’ work time were allocated to documentation and writing (29.3%), communication with ED staff (16.9%) and patients (13.6%). Nurses were engaged in therapeutic and treatment activities (27.6%) and internal communication (17.9%) most of the time. Individual activities were highly fragmented: On average, we recorded 41.3 activities per hour with an average duration of 1.5 min. Nurses had significantly shorter activity durations than ED physicians (F[df = 1] = 4.5, *p* = 0.04). Activity-specific subanalyses revealed differences that could be attributed to professional roles in ED work.

**Conclusion:**

Our results provide reliable and comprehensive insights into the distribution and duration of physician and nurse activities in clinical care in a German ED. Future work and design projects should focus particularly on effects of ED work time allocation on performance and work stress of ED staff as well as on safety and quality of ED patient care.

## Hintergrund und Fragestellung

Arbeitsbedingungen in der klinischen Akut- und Notfallmedizin sind mitverantwortlich für den Arbeitsstress des Personals [1, 2]. Die Studienlage zum tatsächlichen Arbeitsprofil von ärztlich und pflegerisch Beschäftigten ist begrenzt, insbesondere hinsichtlich zuverlässiger Aussagen zur Zeitverteilung, die durch Selbsteinschätzungen weder unter- noch überschätzt werden [3]. Benötigt werden systematische Tätigkeitsanalysen, die die Zeitverteilung des klinisch tätigen Personals zuverlässig beschreiben.

Für Deutschland gibt es nur eine limitierte Zahl aussagekräftiger Studien zu der Zeitaufteilung von Ärzt*innen und Pflegenden auf einzelne Tätigkeiten in der Notaufnahme. In einer Studie in 3 Berliner Notaufnahmen zeigten Mache et al. [4], dass Ärzt*innen zwar 88 % ihrer Zeit in klinischer Arbeit mit Patientenbezug verbringen, diese jedoch in großem Umfang auch Aufgaben der internen Kommunikation, Dokumentation, Meetings und Administration umfasst [4]. Die tatsächliche Arbeitszeit in patientennaher Untersuchung und Diagnostik lag bei 10 % [4]. Ärzt*innen einer pädiatrischen Notfallambulanz waren bis zu 59 % der Zeit in direktem Patientenkontakt, mit ungefähr 20 min pro Patient*in, tätig [5]. Auch Ergebnisse internationaler Studien zu Zeitanteilen direkten Patientenkontakts sind heterogen. Eine Übersichtsarbeit schlussfolgert, dass Fachärzt*innen in der Notfallmedizin von einem Viertel bis zu mehr als einem Drittel der Zeit in direktem Patientenkontakt tätig sind. Bedeutsame Anteile werden für Kommunikation, Dokumentation und administrative Aufgaben aufgebracht. Diese Zeitanteile variieren stark für einzelne Tätigkeiten [6].

Zudem gibt es für den deutschsprachigen Bereich keine systematische Untersuchung, die beide Berufsgruppen vergleichend einbezieht. Dies ist jedoch notwendig, da Ärzt*innen und Pflegende innerhalb des Arbeits- und Versorgungssystems in Notaufnahmen interdependente Aufgaben übernehmen und interdisziplinär kooperieren [7]. Daher ist die Bestimmung berufsgruppenspezifischer Unterschiede und Gemeinsamkeiten essenziell, um eine gesamtheitliche Beurteilung der Zeitverteilung beider Professionen zu gewähren [8, 9]. Von trainierten Expert*innen durchgeführte systematische Beobachtungsstudien („time-motion studies“) sind ein zuverlässiger Weg, Art und Dauer von Aktivitäten der Beschäftigten in der klinischen Akut- und Notfallmedizin zu erfassen [6, 10].

Die Arbeit in Notaufnahmen ist naturgemäß durch Unterbrechungen, Ablenkungen und Multitasking gekennzeichnet [11, 12]. Die fundierte Untersuchung der Anzahl und Dauer von Tätigkeitssequenzen erlaubt Rückschlüsse auf die Fragmentierung von Arbeit [13]. Im Gegensatz zu zahlreichen Studien zu Unterbrechungen und Multitasking in der klinischen Arbeit in Notaufnahmen, fehlen für dieses Setting robuste empirische Befunde für die Anzahl von Aufgabendauern und -wechseln [14, 15]. Häufige Tätigkeitswechsel in der akutmedizinischen Versorgung können Risiken für Fehler oder Beinaheereignisse erhöhen, wenn die kognitive Leistungsfähigkeit, d. h. die Aufmerksamkeit und Gedächtnisleistung des Personals, notorisch überbeansprucht wird [11, 16].

### Fragestellung

1. Welchen Zeitanteil nehmen Aktivitäten im direkten sowie indirekten Patientenkontakt ein (aufgeteilt nach ärztlichem und pflegerischem Personal)?
2. Welche Dauer haben die einzelnen Aktivitäten? Bei welchen Teiltätigkeiten sind häufige Wechsel zu beobachten?

## Methodik

### Design und Setting

Das Studiendesign umfasste standardisierte, 90-minütige Tätigkeitsbeobachtungen bei ärztlichem und pflegerischem Personal einer interdisziplinären Notaufnahme. Diese gehört zu einem süddeutschen Krankenhaus der Maximalversorgung und versorgt im Mittel jährlich 82.000 Patient*innen. Das ärztliche Team besteht aus Internist*innen, Anästhesist*innen, Unfallchirurg*innen und Neurolog*innen, die im 3‑Schicht-System die Notaufnahme durchgehend besetzen. Ärzt*innen anderer Fachabteilungen werden nach Bedarf konsultiert.

Die Notaufnahme umfasst folgende Bereiche: einen internistischen und einen chirurgischen Versorgungsbereich, 2 Schockräume sowie eine Aufnahmestation für Kurzzeitliegepatient*innen („clinical decision unit“). Sie ist eine eigenständige Abteilung des Klinikums mit fest zugeordnetem Personal. Ärzt*innen aus anderen Abteilungen rotieren im Rahmen ihrer Facharztausbildung temporär in die Notaufnahme. Die hier referierten Ergebnisse wurden im Rahmen eines einjährigen Interventionsprojekts zur psychischen Arbeitsbelastung durchgeführt [17]. Es liegt ein positives Votum der Ethikkommission der Medizinischen Fakultät der Ludwig-Maximilians-Universität (Nr. 327-15) sowie des Personalrats des untersuchten Klinikums vor.

### Studienpopulation und Prozedur

Neunzigminütige, standardisierte Beobachtungen pflegerischer und ärztlicher Tätigkeiten wurden während Tagschichten zwischen 09.30 Uhr und 18.00 Uhr in allen oben beschriebenen Bereichen durchgeführt. Einschlusskriterien für Ärzt*innen und Pflegende waren eine regelhafte Beschäftigung in der Notaufnahme, ausreichende Berufserfahrung, um hinreichende Kenntnis der Arbeitsabläufe aufzuweisen sowie eine feste Zuordnung zu einem Arbeitsbereich am Tag der Beobachtung. Studierende im Praktischen Jahr, Praktikant*innen, Auszubildende und Famulant*innen wurden ausgeschlossen.

Die Belegschaft der Notaufnahme wurde vorab durch das Intranet und Vorstellungen in regulären Teambesprechungen über die Studie informiert. Vor jeder einzelnen Tätigkeitsbeobachtung wurde im vorgesehenen Bereich die bzw. der Beschäftigte angesprochen und um die Möglichkeit der Beobachtung ersucht. Nach erfolgter Zustimmung begann die teilnehmende Beobachtung, wobei das Studienpersonal darauf achtete, die Arbeit der bzw. des Beschäftigten nicht zu stören oder zu unterbrechen. Wenn von dem Notaufnahmepersonal oder den betroffenen Patient*innen gewünscht, verließ das beobachtende Studienpersonal den Raum. Es kamen drei Beobachter*innen zum Einsatz, die allesamt vorab zur Anwendung des Instruments trainiert sowie mit dem Umfeld der Notaufnahme vertraut gemacht wurden.

## Methodik

Ein etabliertes und für die Notaufnahme adaptiertes Beobachtunginstrument wurde eingesetzt. Die standardisierte Klassifikation erfolgte in 11 Tätigkeitskategorien, die in Tab. 1 definiert sind [18, 19]. Mittels einer ereignisbasierten Kodierung wurden Beginn und Ende der einzelnen Tätigkeiten als laufende Uhrzeit in Minuten und Sekunden notiert. Voraussetzung für die Kodierung war die erkennbare und dauerhafte Zuwendung des bzw. der Beobachteten zu einer der 11 definierten Teiltätigkeiten.

**Table 1:** 

Tätigkeitskategorie	Teiltätigkeit	Definition
Direkte patientenbezogene Tätigkeit (in direktem Patientenkontakt)	1 Kommunikation mit Patient*innen	Gespräche mit dem bzw. der Patient*in und deren Angehörigen, inkl. Anamnese, Entlassungs‑, Aufklärungsgespräche und sonstigen Themen
	2 Diagnostik	Körperliche Untersuchung sowie alle apparativen und diagnostischen Aktivitäten inkl. Vor- und Nachbereitung, Labor (hier auch Bluttransport)
	3 Therapeutische Aktivitäten/Behandlungsaktivitäten	Ärztliche/pflegerische Behandlung des bzw. der Patient*in i. S. einer Intervention; sonstige Versorgung des bzw. der Patient*in inkl. Vor- und Nachbereitung; Transfer des bzw. der Patient*in
Indirekte patientenbezogene Tätigkeit (in Bezug zu dem bzw. der behandelten Patient*in, aber nicht in direktem Kontakt)	4 Beratung	Kollegialer Austausch zu spezifischen Bedarfen der Patientenversorgung oder zu einzelnen Patient*innen i. S. des diagnostisch-therapeutischen Vorgehens
	5 Dokumentation/Befundung/Schriftarbeit	„Papierarbeit“ mit Patientenakte/-unterlagen; Dokumentation am Computer; Analyse der Laborwerte/Bildgebung etc.; inkl. Transport von Akten
	6 Kommunikation mit Notaufnahmepersonal	Verbaler Austausch mit Kolleg*innen der Notaufnahme mit Bezug zur Patientenversorgung; auch informelle Gespräche mit Kolleg*innen zu Fragen der Patientenversorgung (fachlich, organisatorisch); inkl. Patientenübergaben
	7 Kommunikation mit Anderen (Nicht-NA-Personal)/Telefonate	Verbale Kommunikation mit Personal, das nicht zur Notaufnahme gehört; hierzu zählen auch Telefonate mit externen Anrufer*innen
	8 Organisation/Ablaufkoordination	Aufgaben der Ablauforganisation der eigenen patientenunabhängigen Arbeit und des Arbeits- oder Verantwortungsbereichs, z. B. Dienstplanerstellung
	9 Meeting (regulär, irregulär)	Alle Zusammenkünfte im Team
	10 Lehre/Supervision	Vermittlung von Wissen und Fertigkeiten an Lernende, z. B. Anleitung von Auszubildenden, Instruktion von Studierenden
Anderes	11 Pausen, Erholung, Persönliches	Essen, Privates, Pausen; auch längere persönliche, nicht fachbezogene Gespräche mit Kolleg*innen

Beispiele sind in Tabelle S1 (Zusatzmaterial online) aufgeführt

*NA* Notaufnahme

Die Kodierung der Tätigkeiten erfolgte unmittelbar in der Beobachtung. Lediglich bei unklaren Ereignissen wurden handschriftliche Notizen gemacht, um eine nachträgliche Einordnung ggf. in Rücksprache mit der bzw. dem Beobachteten zu gewährleisten. Für jede Beobachtung wurde die Profession (1 = Pflegekräfte, 2 = Ärzt*innen) notiert. Gleichfalls wurde der jeweilige Versorgungsbereich (1 = konservativ, 2 = traumatologisch, 3 = Aufnahmestation) der Notaufnahme erfasst.

### Statistische Analysen

Kumulierte Zeiten pro Tätigkeit wie auch pro Berufsgruppe wurden zunächst berechnet. Entsprechend der Klassifikation aus Tab. 1 wurden die prozentualen Tätigkeitsanteile patientennaher und -ferner Tätigkeiten aggregiert. Die Raten von Tätigkeitswechseln, d. h. der Wechsel von einer Teiltätigkeit zu einer anderen, wurden für die Gesamtgruppe der Beobachtungen ermittelt. In der Analyse der Einzeltätigkeiten wurden die mittlere Dauer pro Teiltätigkeit wie auch die Anzahl der Tätigkeiten pro Beobachtungsstunde untersucht. Beides wurde für die Gesamtgruppe der Beschäftigten als auch pro Berufsgruppe ermittelt. Die varianzanalytischen Gruppenvergleiche dieser 11 Teiltätigkeiten wurden mit einer α‑Adjustierung nach Bonferroni vorgenommen. Alle statistischen Analysen wurden mit SPSS V24.0 (IBM Inc., Chicago, USA) berechnet.

## Ergebnisse

Insgesamt wurden 160 Einzelbeobachtungen an 40 Tagen durchgeführt, davon 99 bei Pflegekräften (62 %) sowie 61 bei Ärzt*innen (38 %). Die Gesamtzeit aller Beobachtungen betrug 240,8 h (Summe insgesamt: 14.446 min; Beobachtungszeit bei Pflegekräften: 8901 min, Beobachtungszeit bei Ärzt*innen: 5545 min). Die durchschnittliche Dauer einer Einzelbeobachtung betrug 90,3 min (SD = 3,8). Diese Dauer unterschied sich nicht signifikant für Beobachtungen von Pflegekräften und Ärzt*innen (F[df = 1] = 2,61, *p* = 0,11). Es wurden insgesamt 9956 Aktivitäten mit einem Mittelwert von 62,2 Aktivitäten pro Beobachtung kodiert.

Tab. 2 zeigt die prozentuale Verteilung der Zeitanteile der Teiltätigkeiten des pflegerischen und ärztlichen Personals.

**Table 2:** 

Teiltätigkeit	Zeitanteil		
	Gesamt	Pro Berufsgruppe	
		Pflegekräfte	Ärzt*innen
	(%)	(%)	(%)
1 Kommunikation mit Patient*innen	8,8	5,9	13,6
2 Diagnostik	10,3	10,3	10,3
3 Therapeutische Aktivitäten/Behandlungsaktivitäten	19,3	27,6	6,0
4 Beratung	1,1	0,1	2,6
5 Dokumentation/Befundung/Schriftarbeit	19,5	13,4	29,3
6 Kommunikation mit Notaufnahmepersonal	17,5	17,9	16,9
7 Kommunikation mit anderen/Telefonate	7,3	5,9	9,6
8 Organisation/Ablaufkoordination	10,7	13,4	6,3
9 Meetings (regulär, irregulär)	0,8	0,3	1,4
10 Lehre/Supervision	1,0	0,9	1,1
11 Pausen, Erholung, Persönliches	3,7	4,3	2,8

Prozentualer Anteil an der gesamten Beobachtungszeit

**Figure 1:**
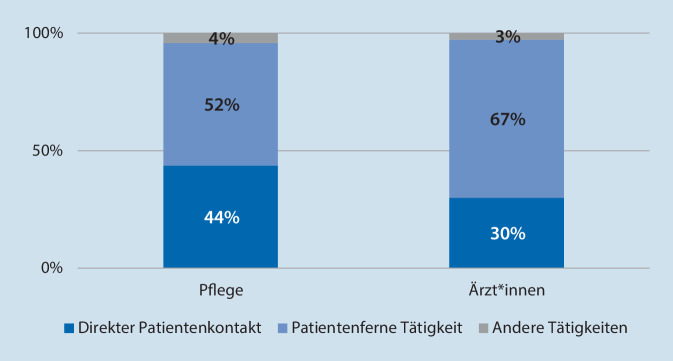

**Figure 2:**
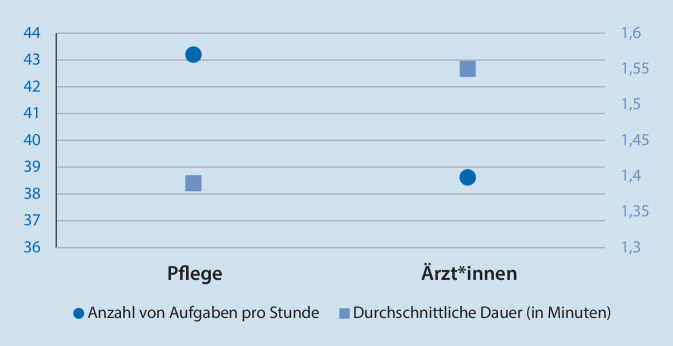

Für die aggregierten Zeitanteile patientennaher und -ferner Tätigkeiten ergaben sich die in Abb. 1 aufgeführten prozentualen Anteile für die Gruppen der beobachteten Pflegekräfte und Ärzt*innen:

Die zweite Forschungsfrage behandelte die Anzahl und Dauer der Einzeltätigkeiten des Notaufnahmepersonals. Insgesamt wurden durchschnittlich 41,3 Tätigkeiten pro Stunde beobachtet (SD = 12,8, Min: 17,3, Max: 106,5). Pflegekräfte (M = 43,0; SD = 14,0) führten im Vergleich zu Ärzt*innen (M = 38,6; SD = 10,3; F[df = 1] = 4,5; *p* = 0,04) eine höhere Anzahl von Tätigkeiten pro Stunde aus (vgl. Abb. 2).

Insgesamt dauerte eine Einzeltätigkeit im Durchschnitt 1,5 min (SD = 1,7). Allerdings war die Gesamtverteilung mit einem Median von 0,95 min stark schief. Insgesamt dauerten 53 % aller beobachteten Tätigkeiten eine oder weniger als eine Minute. Für beide Berufsgruppen ergab sich ein statistisch signifikanter Unterschied mit signifikant kürzeren Aufgabendauern für Pflegekräfte (M = 1,4; SD = 1,5) im Vergleich zu Ärzt*innen (M = 1,6; SD = 1,9; F[df = 1] = 20,7, *p* < 0,01).

Im nächsten Schritt untersuchten wir die Dauer der einzelnen Tätigkeiten bei den beobachteten Berufsgruppen. Tab. 3 weist die über alle Beobachtungen gemittelten Werte aus.

**Table 3:** 

Teiltätigkeit	Durchschnittliche Dauer der Einzeltätigkeiten (in min)				Anzahl von Aufgaben pro Stunde			
	Gesamt	Pro Berufsgruppe		Mittelwertsunterschied	Gesamt	Pro Berufsgruppe		Mittelwertsunterschied
		Pflege	Ärzt*innen	ANOVA		Pflege	Ärzt*innen	ANOVA
	M (SD)	M (SD)	M (SD)	F(df = 1); *p*	M (SD)	M (SD)	M (SD)	F(df = 1); *p*
1 Kommunikation mit Patient*innen	1,2 (1,3)	1,0 (0,9)	1,5 (1,5)	**45,9; <0,001**	4,4 (3,6)	3,7 (3,6)	5,4 (3,4)	**8,5; 0,0042**
2 Diagnostik	1,8 (1,6)	1,8 (1,7)	1,7 (1,6)	0,2; 0,7	3,5 (3,2)	3,5 (3,4)	3,6 (2,9)	0,1; 0,8
3 Therapeutische Aktivitäten/Behandlungsaktivitäten	1,8 (1,8)	1,8 (1,7)	1,7 (1,8)	0,2; 0,7	6,6 (5,5)	9,4 (4,8)	2,2 (3,0)	110,6; **<0,001**
4 Beratung	1,5 (1,1)	0,8 (0,5)	1,5 (1,1)	5,6; 0,02	0,4 (1,0)	0,1 (0,3)	1,0 (1,4)	36,3; **<0,001**
5 Dokumentation/Befundung/Schriftarbeit	1,4 (1,4)	1,1 (0,9)	1,8 (1,8)	**107,5; <0,001**	8,4 (4,3)	7,3 (4,2)	10,0 (4,0)	16,1; **<0,001**
6 Kommunikation mit Notaufnahmepersonal	1,4 (1,8)	1,4 (1,6)	1,4 (1,9)	0,1; 0,7	7,7 (4,0)	7,8 (4,0)	7,5 (4,2)	0,1; 0,8
7 Kommunikation mit anderen/Telefonate	1,1 (0,9)	0,9 (0,8)	1,3 (1,1)	**47,3; <0,001**	4,1 (3,8)	3,9 (4,4)	4,3 (2,6)	0,6; 0,5
8 Organisation/Ablaufkoordination	1,2 (1,1)	1,3 (1,2)	1,0 (0,9)	**9,8; 0,002**	5,4 (4,2)	6,5 (4,7)	3,7 (2,6)	19,2; **<0,001**
9 Meetings (regulär, irregulär)	7,3 (6,1)	4,2 (3,7)	10,0 (6,7)	4,1; 0,1	0,1 (0,2)	0,1 (0,2)	0,1 (0,3)	1,1; 0,3
10 Lehre/Supervision	1,5 (2,0)	1,5 (2,0)	1,4 (1,9)	0,1; 0,8	0,4 (1,0)	0,4 (1,1)	0,5 (1,0)	0,5; 0,5
11 Pausen, Erholung, Persönliches	4,7 (4,8)	4,3 (3,0)	6,0 (8,3)	2,5; 0,1	0,5 (0,7)	0,6 (0,9)	0,3 (0,5)	7,7; 0,006

*N* = 160 Einzelbeobachtungen (*n* = 3547 ärztliche Tätigkeiten, *n* = 6382 pflegerische Tätigkeiten)

**Fett**: *p* < 0,0045 (spaltenweise Bonferroni-Korrektur)

*M* Mittelwert, *SD* Standardabweichung

Die meisten Teiltätigkeiten weisen mittlere Dauern um 1–1,5 min auf. Sehr kurze Sequenzen wurden insbesondere für die Kommunikation mit externem Personal und Telefonate (M = 1,1 min; SD = 0,9), die Organisation und Ablaufkoordination (M = 1,2 min; SD = 1,1) als auch die Kommunikation mit Patient*innen (M = 1,2 min; SD = 1,3) beobachtet. Im Vergleich der Professionen erwiesen sich für Pflegekräfte Kommunikationssequenzen mit Patient*innen, Dokumentationstätigkeiten und externe Kommunikation als signifikant kürzer (vgl. Tab. 3). Für Ärzt*innen waren Organisationsaktivitäten signifikant kürzer im Vergleich zu Pflegekräften.

Abschließend analysierten wir die mittlere Anzahl der Aktivitäten pro Stunde. Für die Gesamtgruppe wurde beobachtet, dass Dokumentation und Schriftarbeit, interne Kommunikation mit Kolleg*innen sowie therapeutische und Behandlungsaktivitäten am häufigsten auftraten (vgl. Tab. 3). Im Vergleich beider Berufsgruppen führten Ärzt*innen signifikant häufiger Kommunikationsaktivitäten mit Patient*innen sowie Beratungen und Dokumentationen aus. Hingegen führte das pflegerische Personal häufiger therapeutische und Behandlungsaktivitäten an Patient*innen durch und es wurden häufigere Aktivitäten der Organisation und Ablaufkoordination beobachtet (vgl. Tab. 3).

## Diskussion

Die fundierte Kenntnis ärztlicher und pflegerischer Arbeitsabläufe erlaubt Rückschlüsse auf die Arbeitsanforderungen in der klinisch-akutmedizinischen Versorgung. Diese Studie stellt erstmalig einen systematischen Einblick in die Tätigkeitsprofile von Pflegekräften und Ärzt*innen in der Patientenversorgung in einer interdisziplinären Notaufnahme vor.

Arbeitszeiten im direkten Patientenkontakt waren bei Pflegekräften umfänglicher als bei Ärzt*innen. Vergleichbare Studien verwenden unterschiedliche Definitionen und Klassifikationen für Tätigkeiten, was einen übergreifenden Vergleich erschwert. Dennoch rangieren unsere Ergebnisse im Rahmen publizierter Studien. Für Notaufnahmeärzt*innen werden Zeitanteile direkten Patientenkontakts zwischen 25 und 40 % berichtet [6]. Dabei ist festzuhalten, dass die angegebenen Werte Schwankungen aufgrund von verschiedenen Aufgabenzuschnitten, Organisations- und Versorgungsformen und -kontexten unterliegen [10, 14, 20]. Insgesamt wird jedoch berufsgruppenübergreifend jeweils weniger als die Hälfte der Arbeitszeit in direktem Patientenkontakt gearbeitet [8, 21, 22].

Unsere Studie liefert zugleich einen Überblick über die Zeitverteilung von Einzeltätigkeiten beider Berufsgruppen. Für Pflegekräfte beobachteten wir in einem Drittel der beobachteten Arbeitszeit direkte therapeutische Arbeit an Patient*innen. Das ist konsistent zu einer älteren, US-amerikanischen Studie, bei der Pflegende im Vergleich zu Ärzt*innen häufiger direkte Aktivitäten an Patient*innen durchführten [8]. Gleichwohl entstammen die wenigen empirischen Vergleichsstudien aus unterschiedlichen nationalen Kontexten. Auch gegenteilige Ergebnisse wurden berichtet, z. Β. dass Pflegekräfte in australischen Notaufnahmen lediglich ein Viertel ihrer Zeit in direktem Patientenkontakt arbeiten [21]. Internationale Vergleiche sind zudem limitiert, da die Aufgaben der Professionen und Zuschnitte der Tätigkeiten sich teilweise deutlich unterscheiden.

Bezüglich Schrifttätigkeiten und Dokumentation beobachteten wir bei Ärzt*innen in unserer Stichprobe einen höheren Zeitanteil als bei Pflegekräften. Ähnliche Ergebnisse wurden auch für dänische Notaufnahmeärzt*innen berichtet, wo ein Drittel der Zeit für Dokumentation aufgewendet wurde [20]. Bei US-amerikanischen Assistenzärzt*innen rangierte dieser Anteil bis zu 50 % der Arbeitszeit, wenn Computer zur Dokumentation und Sichtung patientenbezogener Informationen genutzt wurden [23]. Vergleiche sind wiederum mit Sorgfalt vorzunehmen, da Systeme elektronischer Dokumentation und IT-basierte Anforderungen unterschiedlich implementiert sind [16]. Unsere Ergebnisse unterstreichen zudem die hohe Bedeutung (inter)disziplinärer Kommunikation, die in den Zeitanteilen direkter kommunikativer Interaktion mit Kolleg*innen aus der Notaufnahme und externen Akteur*innen (wie z. B. Sanitäter*innen, Notärzt*innen, Stationspersonal) festzuhalten ist [14, 22]. In beiden Berufsgruppen nimmt die interkollegiale Kommunikation einen bedeutsamen Anteil ein – insbesondere, um aufgabenrelevante Informationen auszutauschen [3, 9].

Unsere Beobachtungen berichten zum ersten Mal für den deutschsprachigen Raum empirisch konsolidierte Befunde zu häufigen Aufgabenwechseln in der Arbeit von Notaufnahmeärzt*innen und -pflegenden. Im Durchschnitt registrierten wir alle 1,5 min einen Tätigkeitswechsel. In der internationalen Literatur wird wiederholt herausgestellt, dass die ärztliche Arbeit in Notaufnahmen mit vergleichsweise kurzen Sequenzen von Tätigkeiten und somit hoher Frequenz von Aufgabenwechseln einhergeht. Gleichwohl ist die empirische Basis belastbarer Studien dazu limitiert [11, 16]. In unserer Erhebung dauerte die Hälfte aller Tätigkeiten weniger als eine Minute. Im Vergleich beider Berufsgruppen identifizierten wir für Pflegekräfte häufigere Aufgabenwechsel, was mit signifikant kürzeren Aufgabendauern einherging. Ein Vergleich mit anderen Studien zeigt, dass die einzelnen, in der Literatur berichteten Raten an Aufgabenwechseln sehr unterschiedlich ausfallen. So berichten z. B. Kee et al. [14] bei australischen Notaufnahmeärzt*innen weitaus höhere Raten (von 66–171 stündlichen Aufgaben bei einer mittleren Rate von 101,4) wie auch Benda et al. bei amerikanischen Notärzt*innen [16]. Jedoch sind die Werte aus Benda et al. deutlich höher als bei vergleichbaren Studien an amerikanischen Notärzt*innen, deren Werte von 33 [8] bzw. 34 stündlichen Aufgabenwechseln [24] näher an den von uns ermittelten Werten für Notaufnahmeärzt*innen liegen.

Zudem weisen unsere Ergebnisse auf berufsgruppenspezifische Unterschiede in der mittleren Dauer und Häufigkeit einzelner Teiltätigkeiten hin. Bei Pflegekräften ermittelten wir sehr kurze Dauern der Patientenkontakte, der Dokumentation wie auch der Kommunikation mit außenstehendem Personal und in Telefonaten. Auch wenn in anderen klinischen Kontexten bereits ähnlich hohe Raten an Aufgabenwechseln für pflegerische Tätigkeiten berichtet wurden, bleiben die individuellen Auswirkungen auf die Aufmerksamkeit, Leistung und den Arbeitsstress sowie auf die Patientensicherheit und Zuverlässigkeit der Versorgung bislang unbestimmt [13]. Bei Ärzt*innen identifizierten wir hingegen kürzere Phasen der Koordination und Ablauforganisation.

### Limitationen

Die Ergebnisse dieser Studie unterliegen Einschränkungen, die bei der Interpretation zu berücksichtigen sind. Teilnehmende Erhebungen weisen inhärente Nachteile von Beobachtungseffekten auf, sodass z. B. beobachtetes Personal Aktivitäten häufiger oder seltener als im Regelfall ausführt. Wir können Selektionseffekte bei der Auswahl der Beschäftigten nicht ausschließen, da die Teilnahme freiwillig war. Wir nutzten einen Ansatz multipler, ereignisbasierter Beobachtungen mit vergleichsweise kürzerer Gesamtdauer von 90 min. Dieses Vorgehen ist der anhaltenden Aufmerksamkeit der Beobachter*innen zuträglich und lässt sich in dem dynamischen Umfeld einer Notaufnahme leichter realisieren [8]. Gleichwohl können Ganzschichtbeobachtungen ein kohärenteres Bild der Arbeitszeit liefern [4]. Diese Studie wurde in einer großen interdisziplinären Notaufnahme eines urbanen Maximalversorgers durchgeführt, was Vergleiche mit kleineren Notaufnahmen in ländlichen Regionen oder solchen mit anderer Organisationsstruktur und anderem Versorgungsangebot einschränkt. Für fächerübergreifende Allgemeinambulanzen sind tendenziell häufigere und kürzere Patientenkontakte berichtet worden als für spezialisierte oder Fachambulanzen [5]. Das Ziel dieser Studie war die Erfassung von klinischen Tätigkeiten in der Akutversorgung, d. h. sog. Frontline-Arbeit. Dadurch sind Tätigkeiten mit hohem Anteil an Führung, Büroarbeit und Administration unterrepräsentiert, wie z. B. die Dienstplanung durch Oberärzt*innen oder Pflegedienstleitungen [25]. Da wir uns auf die Erfassung klar zu bestimmender Tätigkeiten mit direkter Zuwendung von Aufmerksamkeit konzentrierten, sind sehr kurze Tätigkeits- und Interaktionssequenzen unterrepräsentiert bzw. unterschätzt. Zudem wurde ein Tätigkeitsinventar mit generischen Aktivitätskategorien für beide Berufsgruppen genutzt, das mit 11 Teiltätigkeiten vergleichsweise zu grob sein könnte. Inventare mit detaillierterer Aufgliederung einzelner Tätigkeiten bzw. mit spezifischer Aufgliederung für einzelne Berufsgruppen sind verfügbar [6, 18]. Die vorliegenden Ergebnisse berücksichtigen weiterhin keinerlei patientenbezogene Versorgungsbedarfe, wie z. B. Anzahl und Schweregrade der versorgten Patient*innen sowie Personalschichtbesetzungen [21]. Jedoch sind durch die zeitlich verteilten Beobachtungszeiträume wie auch die zufällige Aufteilung der Beobachtungen variante Einflüsse dieser Faktoren limitiert. Nicht zuletzt untersuchten wir nur einen Ausschnitt potenzieller Arbeitsbelastungen in Notaufnahmen [1, 26]. Die Ergebnisse erlauben keine Rückschlüsse auf die Qualität der Patientenversorgung.

### Implikationen für Forschung und Versorgungspraxis

Die Arbeitsbedingungen des Personals und daraus resultierende Belastungen sind kritische Bedingungen für eine effektive und sichere Patientenversorgung in der Notaufnahme [1, 27–29]. Unsere Beobachtungsergebnisse präsentieren eine systematische Bestandsaufnahme der einzelnen Aktivitäten des Personals in akutmedizinischen Bereichen, bedürfen jedoch einer Validierung in anderen Notaufnahmekontexten. Es bleibt zu untersuchen, wie sich unterschiedliche Versorgungsbedarfe und veränderte Rahmenbedingungen auf die beobachteten Tätigkeiten auswirken, z. B. bezüglich einzelner Fachdisziplinen, spezifischer Patientenkollektive oder personeller Besetzungsmuster. In der Fachliteratur wird zudem wiederholt herausgestellt, dass in der akutmedizinischen Arbeit Multitasking eine häufige Strategie ist, um multiple Aufgabenanforderungen zeitnah zu bewältigen [6]. Gleichwohl ist bislang unbeantwortet, welche Verhaltensstrategien Ärzt*innen und Pflegekräfte bei Unterbrechungen oder Mehrfachanforderungen einsetzen, z. B. individuelle Präferenzen für unterbrechende Aufgaben oder individuelle Prioritäten, die Teilaufgaben wie Dokumentation oder Kommunikation eingeräumt werden [15, 30]. Auch der Einsatz von technologischen Systemen der Patientendokumentation und damit einhergehende Anforderungen auf Aufgabenwechsel bleiben größtenteils unbeantwortet; z. B. um in kritischen Phasen resiliente Aufmerksamkeitsleistungen zu ermöglichen [16].

## Schlussfolgerungen

Die klinische Arbeit in Notaufnahmen weist maßgebliche Anteile kommunikativer und dokumentierender Tätigkeiten auf. Für die Gruppe der Pflegekräfte wurden größere Zeitanteile direkter Arbeit an Patient*innen beobachtet. Wir berichten erstmalig für den deutschsprachigen Raum empirisch fundierte Ergebnisse zur Häufigkeit von Aufgabenwechseln in der klinischen Arbeit beider Berufsgruppen. Zukünftige Studien sollten untersuchen, wie die dynamische und fragmentierte Arbeit in Notaufnahmen optimal für die Versorgung von Patient*innen und frei von chronischem Arbeitsstress für die Beschäftigten zu gestalten ist.

## Caption Electronic Supplementary Material


