# Supplementary material for: Time-allocation study of nurse and physician activities in the emergency department
Source: Med Klin Intensivmed Notfmed. 2020 Feb 18;116(3):229–37. [Article in German] doi: 10.1007/s00063-020-00657-4 (PMC8016769; doi:10.1007/s00063-020-00657-4)
Supplement: Supplementary file 1 [file 63_2020_657_MOESM1_ESM.pdf]

Tabelle S1: Beobachtungsinventar pflegerischer und ärztlicher Tätigkeiten in der Notaufnahme (NA, mit Beispielen)

Kategorie	Tätigkeit	Definition	Beispiele
Direkte patientenbezogene Tätigkeit (in direktem physischen Patientenkontakt, „am Patienten“)	**1** Kommunikation mit Patienten	Gespräche mit dem bzw. der Patient*in und deren Angehörigen, inkl. Anamnese, Entlassungs-, Aufklärungsgesprächen und sonstigen Themen	Anamnesegespräch bei Erstkontakt; Patientengespräche zu aktuellem Befinden und Symptomatik, Versorgungssituation und -bedarfen
	**2** Diagnostik	Körperliche Untersuchung sowie alle apparativen und diagnostischen Aktivitäten inkl. Vor- und Nachbereitung, Labor (hier auch Bluttransport)	nähere körperliche Anamnese, EKG, Blut abnehmen, Puls, Labor, auch Bluttransporte
	**3** Therapeutische/ Behandlungs-Aktivitäten	Ärztliche/ pflegerische Behandlung des bzw. der Patient*in, i.S. einer Intervention; sonstige Versorgung des bzw. der Patient*in inkl. Vor- und Nachbereitung; Transfer des bzw. der Patient*in	Nähen einer Platzwunde; Infusion legen, Medikation; auch Transporte von Patienten auf IS usw.
Indirekte patientenbezogene Tätigkeit (in Bezug zu behandeltem Patienten, aber nicht in direktem Kontakt)	**4** Beratung	Kollegialer Austausch zu spezifischen Bedarfen der Patientenversorgung oder zu einzelnen Patient*innen i.S. des diagnostisch-therapeutischen Vorgehens	Abstimmung mit Konsiliarärztin, interdisziplinäre Beratung, Beratung weiteres Vorgehen zwischen Oberarzt und Assistenzarzt (bspw. mit externen Fachärzten)
	**5** Dokumentation/ Befundung/ Schriftarbeit	„Papierarbeit“ mit Patientenakte/-unterlagen; Dokumentation am Computer; Analyse der Laborwerte/ Bildgebung etc.; inkl. Transport von Akten	Informationen zusammentragen, alle patientenbezogenen Dokumentationen, Anordnungen, Arbeit am Computer; Suche/Lesen nach Infos, E-Mailings, OP-Planung, Termine, Info-boards, Schreiben von Arztbriefen/Diktieren von Briefen
	**6** Kommunikation mit NA-Personal	Verbaler Austausch mit Kolleg*innen der Notaufnahme mit Bezug zur Patientenversorgung; auch informelle Gespräche mit Kolleg*innen zu Fragen der Patientenversorgung (fachlich, organisatorisch); inkl. Patientenübergaben	Laboranforderung mit Oberärztin besprechen; ad-hoc Gespräche mit Kollegen aus der NA; hier zählen auch das Fachpersonal NA-nachgeordneter Einheiten dazu (wie Radiologie-/Anästhesie-/Neurologie-Personal)
	**7** Kommunikation mit Anderen/ Telefonate	Verbale Kommunikation mit Personal, welches nicht zur Notaufnahme gehört; hierzu zählen auch Telefonate mit externen Anrufer*innen	Information von Rettungssanitätern auf dem Gang; Gespräch mit Polizei; Arbeitstreffen mit Qualitätsmanagement, usw.
	**8** Organisation/ Ablaufkoordination	Aufgaben der Ablauforganisation der eigenen patienten-unabhängigen Arbeit und des Arbeits- oder Verantwortungsbereiches, z.B. Dienstplanerstellung	Einzelne Abstimmung mit Oberarzt zur Besetzung der Kabinen während der Schicht; Kollegiale Abstimmung bspw. zur Dienstplanerstellung, Diensttausch, etc; Inventuren von Vorräten; Reparatur/Wartung von Gerätschaften.
	**9** Meeting (regulär, irregulär)	Alle Zusammenkünfte im Team	Besprechungen am Morgen oder zu Schichtbeginn; Übergaben bei Schichtwechsel, sonstige Übergaben, Teambesprechungen
	**10** Lehre, Supervision	Vermittlung von Wissen und Fertigkeiten an Lernende, z.B. Anleitung von Auszubildenden, Instruktion von Studierenden	Direkte Erläuterung am Fall für anwesende PJ-Studentin; Einweisung von Pflegeschülerin, Erklärung Sonographiebild für Assistenzärztin
Anderes	**11** Pause, Persönliches	Essen, Privates, Pausen; auch längere persönliche, nicht fachbezogene Gespräche mit Kolleg*innen	Individuelle Zeiten zur Erholung; Toilettengang; persönliche, nicht-fachbezogene Gespräche mit Kollegen; Pausen.
